# A new hope: from neglect of the health sector to aspirations for Universal Health Coverage in Myanmar

**DOI:** 10.1093/heapol/czy110

**Published:** 2019-10-23

**Authors:** Alex Ergo, Thant Sin Htoo, Reena Badiani-Magnusson, Rivandra Royono

**Affiliations:** 1 Broad Branch Associates, Washington, DC, USA; 2 Minister's Office, Ministry of Health and Sports, Nay Pyi Taw, Myanmar; 3 Poverty and Equity Global Practice, The World Bank; 4 Knowledge Sector Initiative, Jakarta, Indonesia

**Keywords:** Myanmar, catastrophic health expenditure, impoverishing health expenditure, equity, health financing, health reforms

## Abstract

Myanmar’s health sector has received low levels of public spending since 1975. Combined with the country’s historic political and economic isolation, poor economic management and multiple internal armed conflicts, these limited resources have translated into low coverage of even the most basic services and into poor health outcomes with wide disparities. They have also resulted in out-of-pocket payments for health as a proportion of total health spending being among the highest in the world. The Government of Myanmar has now affirmed its commitment to moving toward Universal Health Coverage. This commitment is reflected in the National Health Plan 2017–2021. Drawing upon analysis of data from the Myanmar Poverty and Living Conditions Survey 2015 and using the country’s revised methodology to estimate poverty, this paper explores some of the consequences of Myanmar’s excessive reliance on out-of-pocket funding as the main source of health financing. Around 481 000 households in Myanmar experienced catastrophic health spending in 2015. Of this group, 185 000 households lived below the national poverty line. Households that experienced catastrophic health spending spent, on average, 54.7% of their total capacity to pay on health. Of all Myanmar households that went to a health facility in 2015, ∼28% took loans and ∼13% sold their assets to cover health spending. In that same year, ∼1.7 million people fell below the national poverty line due to health spending. The paper then discusses how ongoing reforms could help alleviate the financial hardship associated with care-seeking. With current political will to reform the health system, a conducive macro-economic environment, and the relatively limited vested interests, Myanmar has a window of opportunity to achieve significant progress towards UHC. Continued high-level political support and strong leadership will be needed to keep reforms on track.

**Key Messages**

- Decades of underinvestment in social sectors in Myanmar have led to poor health outcomes and high levels of out-of-pocket spending on health.
- In 2015, ∼1.7 million people fell below the national poverty line due to health spending.
- Of all Myanmar households that went to a health facility in 2015, ∼28% took loans and ∼13% sold their assets to cover health spending.
- The National Health Plan 2017–2021 lays down a strategy to reform the sector and put Myanmar on a path towards Universal Health Coverage.

## Introduction

Myanmar’s health sector has received low levels of public spending for several decades. Combined with the country’s historic political and economic isolation, poor economic management and multiple internal armed conflicts, these limited resources have translated into low coverage of even the most basic services and into poor health outcomes with wide disparities. They have also resulted in out-of-pocket payments for health as a proportion of total health spending being among the highest in the world *(*Ministry of Health and Sports (MoHS) and World Health Organization (WHO), 2017; World Bank (WB), 2018).

The earlier peer-reviewed literature on health financing in Myanmar is sparse, partly reflecting a difficult climate for data collection and analysis. National representation in household survey data was constrained by a 31-year gap between population censuses and by persistent active conflict in multiple states and regions. Despite this difficult analytical context, a few studies have analysed out-of-pocket health expenditures. These can be divided into those focussing on sub-national areas (MoH & WHO, 2008, cited in Sein *et al.*, 2014; Khaing *et al.*, 2015; Mohanty *et al.*, 2017), and those putting forward national-level analyses covering the population outside of conflict-affected areas (Htet *et al.*, 2015; Han *et al.*, 2018). All these studies, however, concentrated on the period prior to Myanmar’s rapid political and economic transition.

This paper aims to address the evidence gap by providing more contemporaneous and nationally representative estimates of the implications of high out-of-pocket spending on health—the need for which was highlighted by Hort (2018)—and assessing the adequacy of current health reforms to address challenges around financial protection. In contrast to earlier publications, the analyses presented in this paper are based on the revised methodology used by Ministry of Planning and Finance (MoPF) and WB (2017a,b) to produce an updated poverty line and poverty estimates based on living conditions in 2015. This paper also provides estimates of the catastrophic payment overshoot and mean positive overshoot to show the intensity of financial hardship.

After providing background on Myanmar’s context, the paper looks into the consequences of the lack of financial protection—in terms of catastrophic and impoverishing spending on health as well as coping strategies adopted by households—drawing upon analysis of data from the Myanmar Poverty and Living Conditions Survey (MPLCS) 2015. It then discusses the ongoing reform process led by MoHS, as embodied in the National Health Plan 2017–2021 (NHP), and assesses how these reforms could help alleviate the financial hardship associated with care-seeking.

## Background

Since 2011, Myanmar has been going through a period of major transition, not only political, but also economic and social. The country has experienced rapid economic growth and has placed renewed attention on the social sectors. From 2013 onward, a discussion around UHC emerged that gained traction among policy makers and health practitioners. The government started signalling a strong commitment to move towards UHC by 2030. The NHP outlines the first phase of Myanmar’s journey towards UHC. Given the country’s starting point, as reflected by current weaknesses in the health system and poor health indicators, achieving the ambitious UHC goals will require substantial efforts and investments.

Decades-long armed conflicts combined with a chronic neglect of Myanmar’s health sector during the military rule have left deep scars. Between 1975 and 2011, the share of health expenditure to gross domestic product (GDP) dropped from 0.8 to 0.3% (WB, 2015). In 2012, government spending on health was among the lowest in the world at around 3% of total government spending, or US$1.6 per capita (WB, 2015). Despite a 5-fold increase in real terms between 2011 and 2015, government spending on health remains extremely low by international standards at a mere 1.1% of GDP (MoHS and WB, 2017).

Prolonged conflicts and the chronic underinvestment in the health sector have led to a gradual deterioration of health service infrastructure, inadequate and unreliable supplies of essential medicines and equipment, and a shortage and maldistribution of health workers, translating into a rapid decline in both the availability and the quality of health services (Sein *et al.*, 2014; Tang *et al.*, 2017). They have also contributed to the country’s poor health outcomes, the wide health inequities and, for the majority of the population, the absence of any form of financial risk protection (Risso-Gill *et al.*, 2014).

Myanmar’s relatively poor health outcomes are illustrated in Table 1, which shows how Myanmar compares to some of the neighbouring countries in terms of selected indicators. At 66.6 years, Myanmar’s 2015 life expectancy at birth was the second lowest among ASEAN countries (WHO, 2018). The gap between the areas with the highest and lowest values within Myanmar is >11 years (Zaw *et al.*, 2015). In 2014, the maternal mortality ratio was estimated at 282 deaths per 100 000 live births (The Republic of the Union of Myanmar, 2016); it ranges from 157 per 100 000 live births in Tanintharyi Region to 357 in Chin State, one of the two poorest states in Myanmar. It is also significantly higher in rural areas than in urban areas (at 310 and 193 per 100 000 live births, respectively) (The Republic of the Union of Myanmar, 2016) and in areas with large proportions of ethnic groups or national races (ADB *et al.*, 2016). With 50 deaths per 1000 live births, under-five mortality in Myanmar remains relatively high for the region (MoHS and ICF, 2017). This indicator as well varies greatly across geographical areas, from 44 deaths per 1000 live births in Mon State to 104 in Chin State. It also varies substantially across socio-economic groups: it is almost four times higher in the poorest wealth quintile than in the best-off quintile (99 deaths per 1000 live births vs 26) (MoHS and ICF, 2017).

**Table 1:** Selected health outcome indicators: Myanmar and other countries in the region

Indicator	Myanmar	Cambodia	Laos	Thailand	Vietnam
Life expectancy at birth (2015)	66.6	68.7	65.7	74.9	76.0
Maternal mortality ratio (modelled estimate per 100 000 live births) (2014^a^ or 2015)	282^a^	161	197	20	54
Under-five mortality rate (per 1000 live births) (2015)	50.0^b^	32.0	66.1	12.6	22.0
Prevalence of stunting among children under the age of five (%) (2015)	29.2^b^	32.4	N/A	10.5	24.6

*Source*: WHO (2018).

a The Republic of the Union of Myanmar (2016).

b MoHS and ICF (2017).

In terms of health care, coverage of even the most essential health services and interventions is generally low in Myanmar and it is highly uneven. For maternal health, for example, the recent Demographic and Health Survey revealed that 60% of births were delivered by a skilled provider and only 37% were delivered in a health facility (MoHS and ICF, 2017). These rates show great geographical variations. In Rakhine State, one of the two poorest states of Myanmar, only 30% of births were assisted by a skilled provider compared with 83% in Yangon Region (MoHS and ICF, 2017). A similar picture emerges for essential reproductive health, child health and nutrition services and interventions, as well as for the diagnosis and treatment of the communicable and non-communicable conditions that account for the biggest disease burden.

One of the reasons for the observed geographical disparities is that many parts of the country controlled by ethnic groups cannot be reached by government services. In many such areas, the population relies solely on basic health services provided by ethnic health organizations (EHOs), non-governmental organizations and/or community-based organizations, many of which lack any government recognition. In many other parts of the county, households tend to rely heavily on private providers, including quacks and drug vendors (Sein *et al.*, 2014). With health providers in the private sector being largely unregulated, households are vulnerable to overpriced services of questionable quality.

Low levels of government funding for health combined with the population’s heavy reliance on private health providers explain why household out-of-pocket payments remain the dominant source of financing for health. In 2015, such payments accounted for 74% of total health spending (MoHS and WHO, 2017). Only five other countries in the world exceeded the 70% threshold.

## Materials and methods

### Data

The analyses presented in this paper draw upon the MPLCS 2015, a living standards measurement survey that collected a wealth of data on the living conditions of Myanmar households between January and April 2015. Beyond being the most recent survey, the MPLCS is also the first expenditures survey to include conflict-affected areas in Myanmar. With a sample size of 3648 households, the survey is representative at the national, urban/rural and agro-ecological zone levels. It also allows analysis by poverty status and expenditure quintiles. The survey includes a few indicators of health status and extensive questions on household expenditures, including expenditures on health, allowing for an assessment of the financial implications of ill-health in the context of overall household spending.

Health-related questions have two recall periods: 30 days and 12 months. Evidence on responses to ill-health can be drawn from both the 30-day module and the 12-month module. The former captures self-reported illness in the last 30 days, and actions that were taken. The latter captures choices of health care providers and health spending. The survey also includes a module on self-reported incidence of shocks and coping strategies, which allows for the analysis of households’ coping strategies to pay for health care.

### Definition of key variables

#### Poverty line

Following a revision of the methodology for measuring poverty, MoPF and WB (2017b) constructed a new national poverty line for Myanmar using the MPLCS 2015 data and based on the needs and living conditions of Myanmar’s population in 2015. This national poverty line, estimated at 1303 kyats (or US$1.27) per adult equivalent in January 2015 prices, was used to produce the estimates presented in this paper. To support global comparisons, the paper also reports results based on the poverty line derived using Xu’s method (Xu, 2004)—estimated at 1990 kyats per adult equivalent per day. Calculations of subsistence spending, capacity to pay, catastrophic spending and impoverishment follow the methods outlined by Xu (2004). Household subsistence spending, the minimum requirement to maintain basic life in a society, can be derived by multiplying the poverty line per adult equivalent by the number of people, in adult equivalents terms, living in the household.

#### Household cash spending

A household’s cash spending is estimated by aggregating the household’s annual monetary spending to purchase or pay for: food, non-food items, and durables, rent, education, and health. A household’s cash spending thus differs from its consumption aggregate in that the latter also takes into account items that are self-produced or received in-kind and imputed rents.

#### Household capacity to pay

A household’s capacity to pay is what the household can spend *on top* of its subsistence spending (Xu *et al.*, 2003). Using the 1990 kyats poverty line, the estimates for household subsistence spending and capacity to pay are 1.6 million kyats (US$1561) per year and 2.2 million kyats (US$2146) per year, respectively.

This definition differs from earlier literature in Myanmar, which has defined capacity to pay using household non-food expenditures (MoH and WHO, 2008; Han *et al.*, 2018) or using the earlier national poverty line (Htet *et al.*, 2015). In contrast, our study uses a measure of capacity to pay calibrated to a global definition to support global comparison, following the methodology outlined in Xu (2004).

#### Household consumption expenditure

A household’s consumption expenditure includes both monetary and in-kind consumption of goods and services, and the monetary value of the consumption of homemade products. Consumption expenditure are used to measure socio-economic position. Households were grouped into economic quintiles (or expenditure quintiles) using consumption expenditure per adult equivalent. The quintiles allow for an assessment of indicators across people living in better- and worse-off households. The quintiles are population weighted implying equal numbers of people in each quintile.

#### Catastrophic health spending

Health spending is considered to be catastrophic if it exceeds some fraction of household resources (e.g. income, expenditure or consumption) in a given period, usually 1 year. The idea is that spending a large fraction of the household budget on health care must be at the expense of the consumption of other goods and services (O’Donnell *et al.*, 2008). This paper looks into households that spend >40% of their capacity to pay on health, as recommended by Xu (2004). Earlier studies in Myanmar also used a 40% threshold to define catastrophic payments (MoH and WHO, 2008; Han *et al.*, 2018). If we apply the earlier definitions of capacity to pay to the MPLCS 2015, estimated catastrophic health expenditure will be higher since capacity to pay is lower when measured using non-food expenditures or the (lower) earlier poverty line.

#### Catastrophic payment overshoot

While catastrophic health spending is a useful measure to get a sense of *how many* households experience financial hardship when seeking care, it does not give any indication of *the degree* or *intensity* of financial hardship. For that, another measure was calculated: the catastrophic payment overshoot, which gives the average degree by which health spending (as a fraction of total cash spending) exceeds the threshold used to calculate catastrophic health spending. It takes the sum of the additional health spending above the threshold for all households that experience catastrophic spending and divides it by the total number of households in the population. A related measure is the mean positive overshoot, which considers the population of households that experience catastrophic spending as the base population.

#### Impoverishing health spending

Health spending is considered impoverishing if the household is pushed below the poverty line as a result of that spending. If the household did not have to spend on health, it could have used the money for other spending, such as food, durables and education. A comparison between poverty estimates that do and do not take into account out-of-pocket spending on health is indicative of the scale of the impoverishing effect of health payments (O’Donnell *et al.*, 2008).

#### Forgone care

Respondents to the MPLCS who reported illness in the last 30 days but did not seek care can be divided into two groups: those who thought that their illness or injury was not serious enough to seek treatment, and all others. Respondents in the latter group are treated as those who forwent care, i.e. those who thought their illness warranted care but who decided not to seek it.

### Data analysis

All data analyses were conducted using Stata statistical software version 14.0. Data were stratified by place of residence (urban/rural), by economic group (expenditure quintile) and by poverty status (above/below the poverty line). Sampling weights were used for all individual and household-level descriptive statistics. Analysis of variance (ANOVA) was carried out to test for significant differences between population subgroups. Monetary values were converted from kyat to US$ using the average exchange rate over the data collection period of 1026 kyat to US$1.

## Results

### Insights into health-seeking behaviour

Use of private health care providers was found to be consistently high across different geographical areas and economic quintiles, with more than half (54%) of those who fell ill in the preceding 30 days seeking care from private providers, including local pharmacies. As shown in Table 2, the proportion was found to be higher in urban areas than in rural areas (70 vs 48%), and among those in the richest quintile than among those in the poorest quintile (65 vs 45%). In urban areas, better-off individuals sought treatment from private health facilities, while those from the poorest quintile relied more heavily on local pharmacies and drug vendors. In rural areas, individuals from poor households made more use of traditional healers, drug vendors, quack doctors and free clinics run by non-government organizations (data not shown).

**Table 2:** Health-seeking behaviour^a^

	Observations (*n*)	Did not seek treatment (%)	Public providers (%)	Private providers (%)	Others (%)
National	5051	21.1	22.6	54.1	2.2
Rural	3239	21.3	27.8	48.3	2.6
Urban	1802	20.6	9.0	69.5	0.9
Poorest quintile	1054	26.4	26.8	44.9	1.9
Best-off quintile	966	17.6	14.4	65.4	2.6

*Source*: analysis of MPLCS 2015 data.

a Of individuals who experienced ill-health in the last 30 days.

The rural population was more likely to visit a public provider than the urban population (28 vs 9%), and so were individuals in the poorest quintile compared with those in the best-off quintile (27 vs 14%).

### The financial burden of health seeking

For the average household in Myanmar, 6.5% of total cash spending was estimated to be used for health-related payments. As shown in Table 3, this share did not vary significantly across economic quintiles and rural/urban locations. In absolute terms, however, variations were considerable. The estimated US$197.5 spent annually on health by the average household (or US$43.6 per capita) ranged from US$121.4 (US$23.2 per capita) for the poorest 40% to US$248.3 (or US$61.1 per capita) for the richest 60%.

**Table 3:** Health spending and forgone care

	Household health spending as share of cash spending (%)	Average household health spending, annual (US$)	Average individual health spending annual (US$)	Share of forgone care in last 30 days (%)
National	6.5	197.5	43.6	4.4
Rural	6.6	156.7	34.8	4.8
Urban	6.2	305.3	66.8	3.5
Poorest 40%	6.8	121.4	23.2	6.0
Best-off 60%	6.3	248.3	61.1	3.4

*Source*: analysis of MPLCS 2015 data.

Rural and poorer households forwent care more often than their urban and better-off counterparts. This is consistent with the finding that cost was the main constraint for the overwhelming majority (83.4%) of those who believed they needed medical attention but did not seek it.

### The hardship associated with health spending

Nearly 1 in 20 households (4.4%) in Myanmar experienced catastrophic health spending in 2015 (see Table 4). This represents around 481 000 households. Of this group, 185 000 households (almost 1 million people) lived below the national poverty line.

**Table 4:** Catastrophic spending on health

	Incidence (proportion of households facing catastrophic spending on health) (%)	S.E. (%)	Overshoot (%)	S.E. (%)	Mean positive overshoot (%)
National	4.4	0.4	0.65	0.09	14.7
Rural	5.1	0.6	0.74	0.40	14.7
Urban	2.7	0.5	0.39	0.08	14.9

*Source*: analysis of MPLCS 2015 data.

The catastrophic payment overshoot was found to be 0.65%. In other words, the average health spending of the entire Myanmar household population exceeded the threshold of 40% of the households’ capacity to pay by 0.65%. The mean positive overshoot was estimated at 14.7%, meaning that households that experienced catastrophic health spending spent, on average, 54.7% of their total capacity to pay on health (i.e. 14.7% higher than the 40% threshold).

In 2015, ∼1.7 million people, or ∼3.4% of Myanmar households, fell below the national poverty line due to health spending. Under the assumption that household expenditures are completely fungible and health care expenditures would have been spent on other items if no household member fell ill, the share of Myanmar’s population who live below the national poverty line increased by 3.2 percentage points (from 28.9 to 32.1%) as a result of households’ out-of-pocket spending on health.

**Figure 1.**
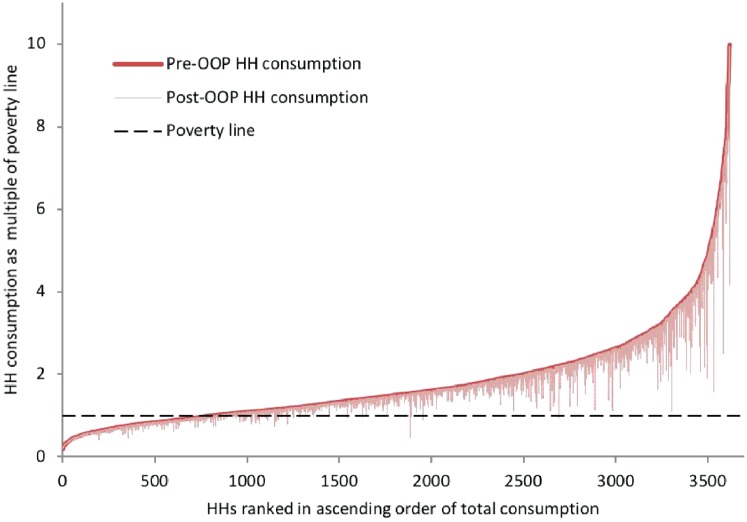
Pen’s Parade of Myanmar’s households, before and after out-of-pocket health spending. *Source*: analysis of MPLCS 2015 data

The impoverishment effect of health spending can be visualized by a ‘Pen’s parade’ of Myanmar’s households (Figure 1). On the *x*-axis, households are ranked based on their total consumption (from poorest on the left to best-off on the right). The *y*-axis provides each household’s total consumption as a multiple of the national poverty line. The solid curved line on the graph indicates each household’s total consumption if they did not spend anything on health. The thin lines that ‘drip’ from the solid curved line show how each household’s total consumption decreases as they are forced to spend on health.

The corresponding figure for the 1990 kyat poverty line is that 1.6 million people were impoverished in 2015 due to out-of-pocket health spending. The share of Myanmar’s population who live below the 1990 kyat poverty line would have dropped from 62.7 to 59.5% if households did not need to pay for their health care out of their pocket. The difference in the impoverished headcounts should be interpreted with caution. While a higher poverty line may give a lower number of individuals falling below the poverty line as a result of their health spending, it also implies that more individuals who were already poor were pushed even further into poverty.

The impoverishing effect of health spending is more pronounced in rural areas than in urban areas. About 4% of the rural population (∼1.5 million people) fell below the national poverty line due to health spending in 2015, compared with only 1.6% of the urban population (∼200 000 people).

### Coping strategies to pay for health care

**Figure 2.**
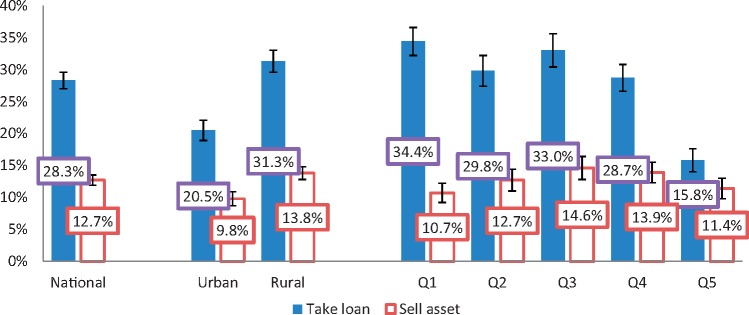
Share of households that took a loan or sold an asset to cover health care expenses*. *Source*: analysis of MPLCS 2015 data; *Of households who made spending on health care in the last 12 months

Many households adopted negative coping strategies to pay for health care, which can have long-term consequences. Of all Myanmar households that went to a health facility in 2015, ∼28% took loans and ∼13% sold their assets to cover health spending. The two coping strategies were not mutually exclusive. These proportions, as well as their breakdowns by type of area and by economic quintile, are displayed in Figure 2. Rural households were significantly more likely to take loans than urban households to deal with health spending, at 28 and 13%, respectively. Between 29 and 34% of households in the lower four quintiles reported to have taken loans to pay for health care. Only in the richest expenditure quintile did the proportion drop significantly to about half that of the other four quintiles (16%). Differences in asset selling behaviour to cover health expenses, on the other hand, were not statistically significant (as shown by the 95% confidence intervals), neither across urban and rural areas, nor across economic quintiles.

## Discussion

The findings presented in this paper provide insights into the adverse effects of out-of-pocket payments in Myanmar. Not only do such payments push many households (or make them sink deeper) into poverty, they also deter many from seeking or adhering to the care they need, leading to persisting health problems and complications, which in turn can lead to an inability to work and earn an income, and to the need for costlier emergency treatment down the road. Finally, they force many households to resort to coping strategies to pay for health care—such as borrowing money or selling assets—which further increase the household’s vulnerability to future shocks. These findings are consistent with those reported by Htet *et al.* (2015).

At 4.4%, the reported rate of catastrophic spending on health is substantially lower than the 14.6% estimated by Han *et al.* (2018), or the figures reported in sub-national assessments [e.g. MoH and WHO (2003) estimate catastrophic expenditures of 34% in urban Yangon]. As discussed in the section on definitions, this partly reflects differences in methodology: the capacity to pay indicator used in this study is higher than that used in earlier studies. It should also be noted that the earlier estimates would have placed Myanmar’s catastrophic out-of-pocket payments as the highest among global estimates, by a substantial margin: Wagstaff *et al.* (2018) estimate the global mean catastrophic out‐of‐pocket payment rate using 40% of non‐food consumption as the threshold to be 3.0% in 2010, with a global high of 12.7%. Similarly, in a multi-country study, Xu *et al.* (2003) find only 2 of the 59 countries included in the study to have a catastrophic spending rate >10% (at 10.1 and 10.5%, respectively).

Both catastrophic and impoverishing spending on health in Myanmar are nevertheless relatively high compared with other countries in the region. In Vietnam, for example, 4.2% of the households faced catastrophic spending on health in 2012 (Hoang *et al.*, 2015), compared with 4.4% in Myanmar. In that same year, an estimated 2.5% of Vietnam’s households were impoverished due to health spending (Hoang *et al.*, 2015), compared with 3.4% of Myanmar’s households.

Narratives from qualitative research suggest that impoverishing expenses tend to be associated with one or more illness events involving hospitalization, repeated care-seeking for unresolved illnesses or a chronic health condition. It is quite common for households to reach the limit of what they can afford to pay after having sought care from several providers, either following the failure of earlier treatments or through subsequent referrals from one provider to another (Save the Children, 2017). Findings from the same qualitative study also reveal how anticipated costs affect households’ health-seeking behaviour. Households’ decision to seek care when a member is sick is predominantly based on their capacity to pay for what they expect to be the cost of medical care and transportation, rather than on their actual health needs. Cash at hand, whether from savings, loans or sale of assets, needs to be secured before a visit to a health provider can be made. In the absence of capacity to pay, households would either forgo care completely or decide to not follow up with referrals even if they perceive that care or follow-up is warranted. What households consider to be their maximum care-seeking and payment is also influenced by the available choice of health care providers and by the perceived likelihood of resolving the health condition. When recovery is seen as unlikely, the household may stop seeking care and treat only immediately life-threatening conditions. Households are then exposed to an additional risk of impoverishment associated with the reduced ability to work and earn an income (for the sick individual and/or for the caregiver) (Save the Children, 2017).

It is worth noting that the estimated rates of forgone care reported in this paper are likely underestimated, especially among the poor, for at least three reasons. First, MPLCS questions on the basis of which the rate of forgone care was calculated referred to a 30-day recall period. The short recall period may have captured a high incidence of mild illness. Out-of-pocket spending required to treat these mild illnesses might not have been prohibitive even for poor households. Second, health-seeking options provided with the questions included local pharmacies, many of which are mere drug vendors, especially in rural areas. Given the definition of forgone care adopted in this article, individuals who went to local pharmacies did not forgo care. However, it is highly conceivable that the sick, especially those from poor households, decided to just treat the symptoms of their illness and not address the underlying health problems. Some portion of those who visited local pharmacies, especially drug vendors, might therefore have in fact decided to forgo the care they needed. Finally, those who did seek care in the 30 days before the survey might subsequently have decided to stop their treatment before the health problem was resolved, or to not follow up with referrals. The analyses presented in this paper could not capture these types of forgone care. Despite the likely underestimation of the rate of forgone care, it is still worth noting that the rate estimated for the bottom two quintiles (6%) is almost twice as high as that among the top three quintiles (3.3%).

If health care payments are extremely high relative to income, households may resort to taking loans or selling assets to finance their health care. This can undermine their livelihood strategies and increase their vulnerability to future shocks (Flores *et al.*, 2008). About a quarter of households in low- and middle-income countries resort to such behaviours (Jamison *et al.*, 2013). The evidence presented in this paper shows how common such coping mechanisms are in Myanmar. It also confirms that more serious (as in the case of hospitalization) or persistent (as in the case of long-term disability or chronic illness) health shocks can be more difficult to deal with financially than frequent, yet smaller, health events. These findings corroborates evidence from earlier studies (Htet *et al.*, 2015; Khaing *et al.*, 2015; Mohanty *et al.*, 2017) and from other countries in the region, such as Thailand (Somkotra and Lagrada, 2009) and Vietnam (Van Minh *et al.*, 2013; Mitra *et al.*, 2016).

Myanmar’s excessive reliance on out-of-pocket payments as a source of health financing affects not only the health and wellbeing of individuals and households, especially the poor and vulnerable; it also impedes societal development more broadly, by perpetuating poverty, hampering economic growth—poor health reduces worker productivity and increases absenteeism—and exacerbating inequities.

The main underlying causes for the disproportionate share of out-of-pocket payments in the country’s total spending on health are known. They include:

- **‘The government’s chronic underfunding of the health sector’**, which has led to a weak health system, a lack of availability of even the most essential health services and generally poor quality of existing services.
- ‘The weak oversight of the private sector’, resulting into a wide array of unregulated informal health care providers, from quacks to drug vendors and unregistered clinics, often selling sub-standard or counterfeit drugs and providing ineffective care.
- ‘The absence of large-scale pre-payment and pooling arrangements’ to distribute risks amongst the population and mitigate the financial impact of episodes of care.
- ‘The decades of armed conflict’, which have further isolated large groups of the population, depriving them from basic services.

The remainder of this section describes Myanmar’s ongoing efforts to address these underlying causes and discusses the work that still lies ahead.

### Towards a brighter future: health reforms with a vision and an engine

After many decades of isolation under the military rule, Myanmar is slowly opening up. The country benefits from a conducive macro-economic environment, with a growth rate projected to be around 7% in the medium term (WB, 2017). Social sectors, which have long been neglected, are receiving renewed attention. The country’s leaders recognize the critical role of a healthy and educated society for the sustainable development of the country and they are determined to turn the tide. Their commitment to the UHC goals is manifested by the NHP, which represents a major shift away from business-as-usual, and by the establishment of a dedicated unit under the Minister’s Office—the NHP Implementation Monitoring Unit (NIMU)—to orchestrate and monitor the plan’s implementation.

The NHP aims to strengthen the country’s health system and lay the foundation for Myanmar’s journey towards UHC, adopting a path that is explicitly pro-poor. Its main goal is to extend access to a basic ‘Essential Package of Health Services’ (EPHS) to the entire population while gradually increasing financial protection. The basic EPHS emphasizes the critical role of primary health care and the delivery of essential services at the Township level and below, starting within the community. This represents an important shift in focus considering how, for many decades, the health budget has been skewed towards medical care provided in hospitals and towards urban areas (MoHS, 2016).

Some of the key elements of the strategy outlined in the NHP are the substantial phased investments in supply side readiness at Township level and below; the engagement of non-MoHS health care providers, including private-for-profit GP clinics, EHOs and non-governmental organizations; the strengthening of the health system; and the move from top-down planning to inclusive bottom-up planning to make the system more responsive to the local needs. Global evidence lends its support to this strategy. First, the definition of a relatively modest explicit benefit package (the basic EPHS), that everyone should have access to by the end of the NHP period and that will subsequently grow to reflect Myanmar’s increased fiscal space for health and its expanded capacity to deliver quality services, is considered to be essential for the sustainability of a health system (Cotlear *et al.*, 2015; Glassman *et al.*, 2016). Second, the strong emphasis, within the benefit package, on primary health care is in line with global evidence showing that functioning primary care is a cornerstone of UHC (Bump *et al.*, 2016; Stigler *et al.*, 2016). Third, strengthening of the health system to support effective delivery of quality services is recognized as a means to progress towards UHC (Bump *et al.*, 2016; International Health Partnership UHC2030, 2017). Fourth, the importance of adopting a pro-poor path to UHC—a principle referred to as ‘progressive universalism’ (Gwatkin and Ergo, 2011)—is now widely recognized (Jamison *et al.*, 2013; WHO, 2014; Cotlear *et al.*, 2015; Bump *et al.*, 2016). Finally, the benefits of recognizing and engaging EHOs extend beyond health; recognition and engagement encourage frequent dialogue and stimulate closer collaboration, which in turn can benefit the broader peace process (Jolliffe, 2014; Davis and Jolliffe, 2016; Tang and Zhao, 2017).

Bringing essential services closer to the communities and strengthening the health system to make sure that those services can be delivered effectively should, at least in theory, contribute to reducing households’ out-of-pocket expenditures in a number of ways:

- Confidence that services delivered at public facilities are available and are of quality will gradually reduce the perceived need to seek care from informal private providers.
- The availability of services closer by will shorten delays in seeking and accessing care.
- The improved effectiveness of services will result in fewer complications and fewer persisting health problems.
- If necessary drugs and medical supplies are available at the public facility, patients will no longer need to buy them outside the facility.
- Households will need to spend less on transportation and they will regain their ability to work and earn an income faster.

This is only part of the picture, however. Substantial improvements in financial protection will only be possible if some form of risk pooling is introduced. Moreover, all of the above will only be achieved if additional resources for health can be mobilized while existing resources are used more efficiently. This supports recommendations made by Sein *et al.* (2014).

### A health financing strategy: the chapter yet to be written in the reform agenda

The NHP recognizes the importance of a coherent health financing strategy for achieving the country’s UHC goals. The formulation of such strategy is ongoing. The outcome will largely determine the extent to which Myanmar’s chosen path towards UHC is truly pro-poor and whether a meaningful increase in financial protection can be expected in the foreseeable future. The strategy will specify where the money will come from to finance the required expansion of service delivery and strengthening of the health system. It will also clarify how risks will be pooled to provide adequate financial protection. Finally, it will stipulate how these resources will be allocated and how services will be purchased, and by whom, to ensure equitable coverage of the benefit package (Bump *et al.*, 2016; Kutzin *et al.*, 2017).

With respect to resource mobilization, there is growing consensus globally that predominant reliance on compulsory or public financing is essential for UHC (Kutzin, 2012; Bump *et al.*, 2016). In the context of Myanmar, public financing will be particularly important for at least two reasons. First, 32% of the population lives below the poverty line, and another 14% is considered near-poor (MoPF and WB, 2017b). Second, >75% of the Myanmar labour force is engaged in work in the informal sector (Department of Labour, 2016). Collecting compulsory contributions from this group will be extremely challenging (Bitran, 2014; WHO, 2015).

In terms of risk pooling, Myanmar does not have to date a comprehensive health insurance system. Apart from a social security scheme that covers <2% of the population, the *de facto* pool for the vast majority is the government health budget, which in 2015 amounted to only US$12 per capita (MoHS and WHO, 2017). There is still an opportunity for Myanmar to move towards a single risk pool and achieve optimal cross-subsidization (Kutzin, 2012).

With respect to the purchasing function, the NHP recognizes that in-country experience in strategic purchasing is extremely limited. A few pilot initiatives involving the contracting of non-MoHS providers by an entity that simulates the role of a purchaser (Crapper *et al.*, 2017) are already generating valuable lessons. The skills built through these initiatives will subsequently be transferred to a designated purchasing entity that will expand, replicate and adapt similar purchasing arrangements with the different types of providers while further strengthening the key functions of a purchaser. It will be critical to simultaneously address the numerous bottlenecks associated with the rigid and largely outdated public financial management rules.

### A strong plan: necessary but insufficient

While important, a sound plan is only the beginning. What matters now is the actual implementation of that plan. The establishment of a dedicated unit, NIMU, to be the engine behind the reforms increases the odds of success. Yet, it does not reduce the number or lessen the magnitude of the challenges that stand in the way. Some of these challenges are noteworthy. First, mistrust in government runs deep, not only among the general population but even more so among ethnic groups and EHOs (Davis and Jolliffe, 2016). Second, despite the signing of a national ceasefire agreement by many of the ethnic armed groups, ∼118 of Myanmar’s 330 townships are still affected to some extent by conflict (The Asia Foundation, 2017), hampering efforts to deliver basic health services to populations living in those areas. Third, the long disengagement from social sectors has also affected the strength of some of the institutions at the different levels of the health system that are key to the successful implementation of the reforms. Limited management capacity and weak governance in particular represent major barriers. Finally, moving away from business as usual requires a real shift in mindset, incentives and institutional culture. Such a shift does not happen overnight, especially when it involves breaking habits that have been nurtured for many years. Getting all stakeholders to align behind a common plan and getting them to work together on the plan’s implementation is extremely challenging.

## Conclusion

With current political will to reform the health system, a conducive macro-economic environment, the relatively limited vested interests blocking the pathway to reform, and a population thirsty for meaningful change in the provision of affordable quality health services, Myanmar has a window of opportunity to act and achieve significant progress towards UHC. Continued high-level political support and strong leadership will be needed to keep the reforms on track and to strengthen the institutions that are key to their successful implementation.
